# Structural Alterations of Antigens at the Material Interface: An Early Decision Toolbox Facilitating Safe-by-Design Nanovaccine Development

**DOI:** 10.3390/ijms221910895

**Published:** 2021-10-08

**Authors:** Litty Johnson, Lorenz Aglas, Wai Tuck Soh, Mark Geppert, Sabine Hofer, Norbert Hofstätter, Peter Briza, Fatima Ferreira, Richard Weiss, Hans Brandstetter, Albert Duschl, Martin Himly

**Affiliations:** Department of Biosciences, University of Salzburg, 5020 Salzburg, Austria; litty.johnson@sbg.ac.at (L.J.); lorenz.aglas@sbg.ac.at (L.A.); waituck.soh@sbg.ac.at (W.T.S.); mark.geppert@sbg.ac.at (M.G.); sabine.hofer@sbg.ac.at (S.H.); norbert.hofstaetter@sbg.ac.at (N.H.); Peter.Briza@sbg.ac.at (P.B.); fatima.ferreira@sbg.ac.at (F.F.); richard.weiss@sbg.ac.at (R.W.); hans.brandstetter@sbg.ac.at (H.B.); albert.duschl@sbg.ac.at (A.D.)

**Keywords:** Alhydrogel^®^, silica nanoparticles, Bet v 1, BM4, SiO_2_, allergen, structural integrity, alum

## Abstract

Nanomaterials have found extensive interest in the development of novel vaccines, as adjuvants and/or carriers in vaccination platforms. Conjugation of protein antigens at the particle surface by non-covalent adsorption is the most widely used approach in licensed particulate vaccines. Hence, it is essential to understand proteins’ structural integrity at the material interface in order to develop safe-by-design nanovaccines. In this study, we utilized two model proteins, the wild-type allergen Bet v 1 and its hypoallergenic fold variant (BM4), to compare SiO_2_ nanoparticles with Alhydrogel^®^ as particulate systems. A set of biophysical and functional assays including circular dichroism spectroscopy and proteolytic degradation was used to examine the antigens’ structural integrity at the material interface. Conjugation of both biomolecules to the particulate systems decreased their proteolytic stability. However, we observed qualitative and quantitative differences in antigen processing concomitant with differences in their fold stability. These changes further led to an alteration in IgE epitope recognition. Here, we propose a toolbox of biophysical and functional in vitro assays for the suitability assessment of nanomaterials in the early stages of vaccine development. These tools will aid in safe-by-design innovations and allow fine-tuning the properties of nanoparticle candidates to shape a specific immune response.

## 1. Introduction

Engineered nanomaterials have gained significant research interest as novel vaccination platforms. They have been utilized to enhance vaccine efficacy by modulating the quality, specificity and durability of immune responses towards a specific antigen [1]. Adjuvants and/or carriers are important in vaccine formulations as they are essential for the efficient activation of the immune system. In this regard, nanomaterials are attractive platforms as they can transport protein antigens effectively close to or even into antigen-presenting cells, leading to decreased systemic or local side effects concomitant with a reduction in the antigen dose [2], in addition to their intrinsic immunomodulatory properties [3,4]. Moreover, the huge diversity in the physicochemical properties of engineered nanomaterials along with their ability to be tailored can be harnessed for directing a specific immune response [5]. Together, these novel opportunities have contributed to an explosion in the exploration of nanoparticles (NPs) in novel vaccine technologies, especially in the field of infectious disease, allergy and cancer [6,7,8].

However, despite their promising potential, only a limited number of nanovaccines have been approved for clinical use thus far. This may be due to challenges in the clinical translation and commercialization of nanomaterials, which include restrictions in large-scale manufacturing, establishment of biopharmaceutical correlations between the properties of the nanomaterial and their in vivo performance and the lack of clear regulatory and safety guidelines [9]. The structural and physicochemical diversity of nanomaterials complicates large-scale manufacturing and can pose difficulties for quality assessment [10]. Compared to other biopharmaceutical products (e.g., highly purified monoclonal antibody preparations), vaccines are, in general, more complex due to the often heterogeneous nature of the active components (live-attenuated or inactivated/chemically modified whole microbes, crude allergen extracts, etc.) and their interaction with adjuvants or other excipients [11]. This contributes to increased concerns regarding the safety and efficacy of vaccines and results in the scrutiny of novel vaccine technologies. To a great extent, vaccines still rely on in vivo testing; nevertheless, a strong demand for novel in vitro testing methods ensuring consistency of vaccine formulations has been articulated [12]. The establishment of in vitro testing methods which are applicable early in the development pipeline and have a satisfying predictive power is in high demand. These methods in combination with functional assays can be used to determine the safety, efficacy and consistency of nanovaccines.

One critical step in the development of nanovaccines includes the rational selection of a nanoparticulate adjuvant for a specific antigen aiming at eliciting a desired immune response. To attain a potent antitumor response, a nanoparticulate system that can enhance the host immunity is favored, whereas in the case of hypersensitivity or autoimmune disorders, materials suppressing a harmful immune response are beneficial [13]. Thus, tailoring the physical and biochemical properties of NPs is crucial in evoking a specific immune response, thereby improving the efficacy of vaccines with an optimum safety profile. Tailoring novel vaccine strategies is of particular interest for the elderly and immunocompromised population, who are more prone to diseases and less responsive to vaccines [14]. In the case of the SARS-CoV-2 pandemic, we can see a direct impact of age on the increased vulnerability to severe infection [15]. The increased susceptibility to infection and diminished responsiveness to vaccines can be explained by the reduced number of naïve T cells, reduced T cell receptor diversity and other defects in the innate immune cells usually associated with aging [16]. Hence, to increase the efficacy of vaccines in the immunocompromised or elderly population, NPs that can induce inflammatory responses in a well-controlled fashion may be favorable as they are reported to enhance T cell function [17]. Furthermore, the tunable properties of NPs can be used to manipulate and optimize the quantity and proteolytic accessibility of antigens [18]. The differences in an antigen’s stability, spacing and orientation when linked to NPs can affect the immune response. For example, a desired antibody response with negligible adverse effects can be accomplished by intentionally masking the undesirable epitopes of the antigen and exposing the desired epitopes. Once the structural integrity of an antigen associated with a specific NP is established, it may be tuned in such a way that a desired outcome can be achieved. By understanding its structural integrity, we can predict the behavior of an antigen upon conjugation to NPs. The resulting insights in antigens’ immunogenicity will ultimately facilitate a “safe-by-design” nanovaccine development strategy [19].

In this study, we established an in vitro test strategy to study the interaction of nanoparticulate adjuvants with antigens, investigating the structural integrity of antigens at the nanoparticulate surface by using biophysical methods and a set of functional assays. We aimed to evaluate the early events in immune modulation to fine-tune nanoparticulate adjuvants. The goal was to test for desired immunological outcomes of novel nanovaccines displaying an optimal safety and efficacy profile. SiO_2_ NPs were used as a model nanoparticulate platform and were compared with Alhydrogel^®^, the most-applied particulate adjuvant in vaccines. Alhydrogel^®^ or alum is still considered as the gold standard among the clinically available adjuvants despite its recognized drawbacks [20,21]. SiO_2_ NPs, on the other hand, are promising candidate adjuvants and/or vaccine carriers and are widely studied due to their biodegradability, biocompatibility, ease of surface modification, low production costs and low toxicity [22]. The model proteins used for this study included Bet v 1, the major birch pollen allergen, and BM4, a molten globule-like hypoallergenic variant of Bet v 1 [23]. Both proteins display almost identical amino acid sequences (with a deviation in five amino acids only) but differ in their conformational stability [24]. We deliberately opted for model allergens with different conformations to determine if the conformational stability of the antigens has an influence on the interaction of the proteins and the particulate systems. The model proteins were conjugated to both selected particulate systems by non-covalent surface adsorption. This is the best established approach in developing licensed particulate vaccines due to its simplicity and increased effectiveness [25].

## 2. Results and Discussion

### 2.1. Characterization of the Synthesized SiO_2_ NPs and Alhydrogel^®^

**Figure 1:**
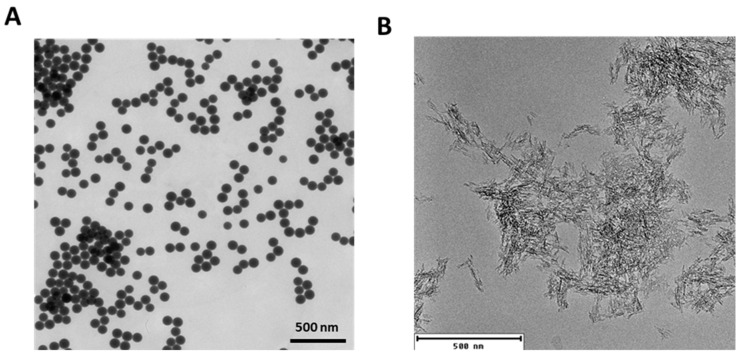
Characterization of the particulate systems by determining the morphology and primary size of particles. Transmission electron microscopy (TEM) image of synthesized SiO_2_ NPs (**A**) and Alhydrogel^®^ (**B**).

As the initial step, we characterized the two particulate systems to determine differences in their physicochemical properties. We adopted the microemulsion method for the synthesis of SiO_2_ NPs as this method has been reported to produce highly monodisperse NPs [26]. Characterization of the synthesized NPs by dynamic light scattering (DLS) indicated a highly monodisperse suspension of SiO_2_ NPs with an average hydrodynamic diameter of 100.3 ± 3.4 nm and a zeta potential of −38.9 ± 2.8 mV. Consistently, monodispersity and a hydrodynamic diameter of 102.4 ± 39.3 nm were confirmed by nanoparticle tracking analysis (NTA) (Table 1). Furthermore, from the transmission electron microscopy (TEM) images, the primary particle size of the synthesized NPs along with the uniform spherical shape was further established. The average diameter of the NPs was calculated from the TEM images and was found to be 96.3 ± 4.9 nm (Figure 1A). On the contrary, Alhydrogel^®^ was found to be highly polydisperse, with an average size of 585.9 ± 174.2 nm, and exhibited a surface zeta potential of +18.0 ± 1.5 mV (Table 1). Alhydrogel^®^ consisted of large clusters of nanofibers, which heavily agglomerated to form microstructures, as evident from the TEM images (Figure 1B and Figure S1A). The scanning electron microscopy (SEM) images further confirmed these heterogeneous microstructures (Figure S1B). The presence of these larger agglomerates impaired their characterization by NTA. The characterization data for Alhydrogel^®^ concur with previously published reports [27,28]. From these characterization data, the morphological distinction of both particulate systems was evident.

### 2.2. Efficient Conjugation of Allergens with SiO_2_ NPs and Alhydrogel^®^

**Figure 2:**
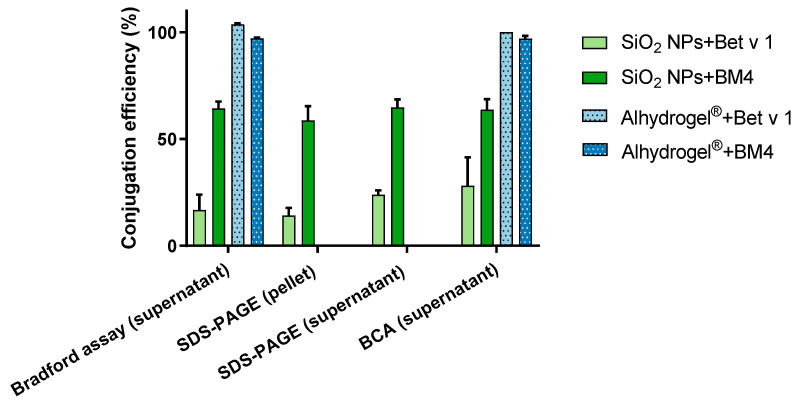
Conjugation efficiency of particulate systems. The quantitative analysis of percentage of allergen bound to SiO_2_ NPs and Alhydrogel^®^ was performed by SDS-PAGE, Bradford assay and BCA assay. A 100% conjugation efficiency here represents a conjugation of the total amount of allergen taken for the experiment (160 µg/mL).

Following the characterization of particles, we examined their conjugation efficiency with the model allergens. The allergens were incubated with the particles, and the conjugated allergens were quantitatively and qualitatively analyzed. From the analysis of the centrifuged pellets by sodium dodecyl sulphate-polyacrylamide gel electrophoresis (SDS-PAGE), a conjugation efficiency of 14.5 ± 3.5% for Bet v 1 and 58.7 ± 5.8% for BM4 was obtained with SiO_2_ NPs (Figure 2). To further confirm our observation, the supernatant, which contained the non-conjugated allergen, was quantified using standard protein assays such as Bradford and bicinchoninic acid (BCA) assays. The amounts of protein bound were back-calculated from the concentrations obtained from these assays. From the Bradford assay, the percentage of conjugation efficiency was found to be 16.8 ± 6.2% for Bet v 1 and 64.3 ± 1.5% for BM4. This was similar in the BCA assay, where 28.1 ± 11.8% of Bet v 1 and 63.7 ± 4.8% of BM4 were conjugated to SiO_2_ NPs (Figure 2). These results are consistent with the SDS-PAGE analysis. Thus, we observed significant differences in conjugation efficiency with both allergens when bound to SiO_2_ NPs. This discrepancy can be attributed to the conformational differences in both allergens [24]. We used only BCA and Bradford assays for the quantification with Alhydrogel^®^ as we obtained comparable results from all three methods using SiO_2_ NPs, and a lack of complete dissociation of allergens from Alhydrogel^®^ leads to technical difficulties in their analysis by SDS-PAGE. Alhydrogel^®^ exhibited a conjugation efficiency of 103.7 ± 0.4% for Bet v 1 and 97.0 ± 0.3% for BM4 from the Bradford assay. Similarly, we attained 99% efficiency with both allergens from the BCA assay (Figure 2). Furthermore, the conjugation efficiency of both candidate allergens and the particles was confirmed qualitatively by the changes in the zeta potential. Upon conjugation of the allergens to SiO_2_ NPs or Alhydrogel^®^, we observed a more positive zeta potential compared to the pristine NPs (Table 2). This further confirms the effective conjugation of the allergens. From all these data, it can be concluded that the conjugation efficiency of allergens with both candidate NPs is quite different. From previous reports, Alhydrogel^®^ adsorbs proteins dominantly through ligand exchange and by electrostatic interactions [29], whereas SiO_2_ NPs adsorb proteins mainly by hydrophobic and electrostatic forces [30,31]. As both model allergens have a pI of around 5 (Bet v 1, 5.4; BM4, 5.6), they exhibit a negative net charge at pH 7.4 [32]. Alhydrogel^®^ displays a strongly positive zeta potential, which further indicates the possible role of strong electrostatic interactions leading to the increased conjugation efficiency in Alhydrogel^®^. Furthermore, the network of nanofibrillary structures of Alhydrogel^®^ can create more binding sites for the protein compared to the highly monodisperse spherical SiO_2_ NPs used as model NPs here (Figure 1). SiO_2_ NPs exhibit a negative zeta potential at neutral pH. Thus, theoretically the role of electrostatic interactions should be negligible or repulsive in SiO_2_ NPs. However, there are reports indicating the adsorption of proteins to equally charged surfaces [33]. Even though a protein exhibits an overall negative charge, it can have local positively charged surface patches [34]. These areas in the allergen can attract the negatively charged SiO_2_ NPs, leading to weak attractive electrostatic interactions resulting in effective protein conjugate formation. 

### 2.3. Conjugation of Allergens Decreases Their Proteolytic Stability 

**Figure 3:**
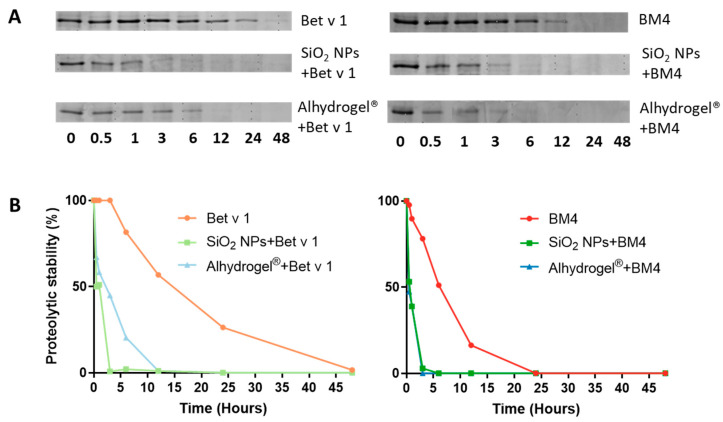
Impact of allergen conjugation to particles on the kinetics of allergen processing. (**A**) Comparison of the processing behavior of allergens conjugated to the particulate systems and the unconjugated allergens by assessing their proteolytic degradation at 37 °C for different time points (0, 0.5, 1, 3, 6, 12, 24 and 48 h) using the microsomal extracts from JAWS II determined by 15% SDS-PAGE and Coomassie staining (left panel denotes samples with Bet v 1 and right panel those with BM4), and (**B**) densitometric analysis of their proteolytic stability with Image lab 4.01 software (left panel denotes samples with Bet v 1 and right panel those with BM4).

**Figure 4:**
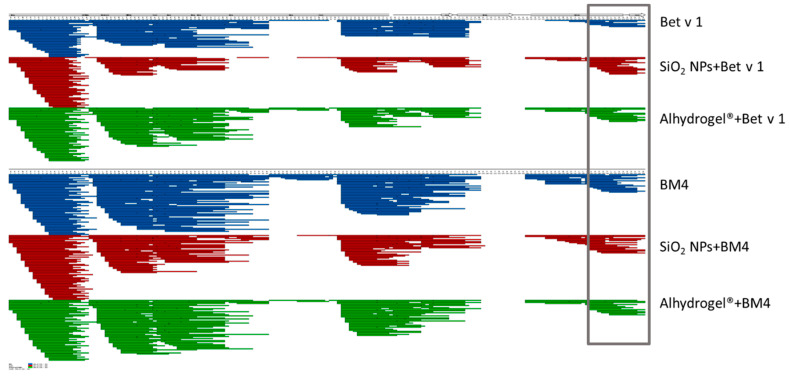
Impact of allergen conjugation to particles on the outcome of allergen processing determined qualitatively. The peptides obtained after 1 h of proteolytic degradation at 37 °C by LC-MS using the microsomal extract from JAWS II cells are displayed. The immunodominant T cell epitope region is highlighted in gray.

**Figure 5:**
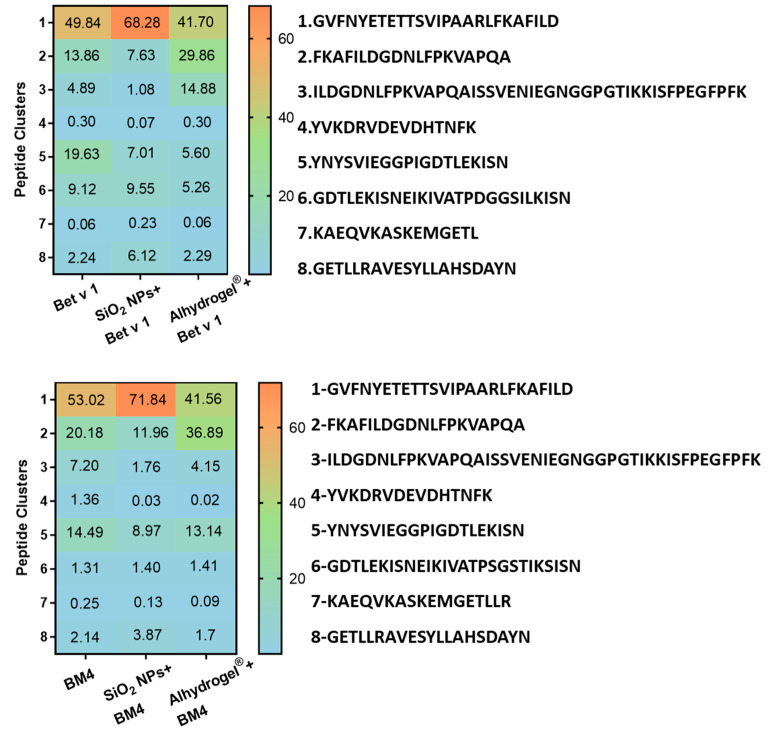
Impact of allergen conjugation to particles on the outcome of allergen processing determined quantitatively. Relative abundances of peptides obtained after 1 h of proteolytic degradation using the microsomal extract from JAWS II cells are shown (upper panel indicates samples with Bet v 1 and lower panel those with BM4). The peptides obtained by LC-MS analysis were grouped into eight clusters based on the qualitative data.

The structural stability of proteins can affect the major molecular events leading to an immune response, from uptake to antigen presentation, thereby exerting a significant impact on the immunogenicity of the administered vaccine [35,36]. As an initial method to understand the structural integrity of the conjugated protein, we determined the proteolytic stability of the allergens conjugated to SiO_2_ NPs and Alhydrogel^®^ by comparing the degradation kinetics, using microsomal extracts of an immortalized murine dendritic cell line (JAWS II). The microsomal extracts are composed of several exo- and endoproteases which include cathepsin S, D, K and L and legumain, amongst others [37]. These proteases proteolytically process the protein antigens to smaller peptides for their presentation by MHC molecules. Initially, we confirmed that the conjugation of SiO_2_ NPs and Alhydrogel^®^ did not inhibit the activity of proteolytic enzymes by an enzymatic assay (Figure S2). Furthermore, the stability of allergen–NP conjugates in the microsomal degradation assay was verified (Figure S3). We observed that conjugation of allergens to both candidates increased the proteolytic susceptibility of the allergen. Bet v 1 bound to SiO_2_ NPs and Alhydrogel^®^ was proteolytically cleaved much faster than the unconjugated allergen (Figure 3A,B). With BM4, we observed a similarly increased degradation upon conjugation (Figure 3A,B). This observation was further confirmed by simulating in vitro endolysosomal processing using recombinant human cathepsin S, a prominent endolysosomal cysteine protease (Figure S3). In accordance with the former results, a similar trend in the kinetics of degradation was evident. Subsequent analysis of the peptides derived from the endolysosomal degradation using liquid chromatography-mass spectrometry (LC-MS) resulted in qualitatively identical degradation patterns (Figure 4). However, we could observe a large diversity in the peptides generated for the conjugated samples. Interestingly, alterations in the kinetics of proteolytic processing also prompted the generation of more peptides in the immunodominant T cell epitope region [38] (Figure 4, highlighted in gray). For the quantitative analysis of the peptides, we grouped the generated peptides into eight main clusters based on the qualitative data and calculated the percentage of relative abundances for each peptide cluster (Figure 5). The conjugation of allergens to both particle systems clearly affected the rate of peptide production in different ways. Conjugation of both allergens to SiO_2_ NPs significantly increased the relative abundance of peptides in clusters 1 and 8 compared to the unconjugated allergen. However, in Alhydrogel^®^, we observed this shift towards peptide clusters 2 and 3 (Figure 5). The increased rate of core peptide production in cluster 8, thus, resulted in a more efficient generation of the immunodominant T cell epitope in the case of SiO_2_-conjugated allergens. However, the increased or decreased peptide abundance in other clusters was, immunologically, not as significant as cluster 8. Even though both particles showed identical processing patterns, there is a deviation in their relative abundance of peptides. The fold or conformational stability of allergens has previously been shown to have a huge impact on the processing and immunogenicity of allergens [39]. Thus, the increased proteolytic processing of allergens upon conjugation to particles may be due to a change in the conformational stability of the allergens, and the deviation in the relative abundance of peptide clusters may indicate alterations in the interaction of allergens with the two different particulate systems, leading to differences in preferential exposure to the proteolytic enzymes. 

### 2.4. Changes in the 3D Fold of the Allergen upon (Nano)Particle Conjugation

**Figure 6:**
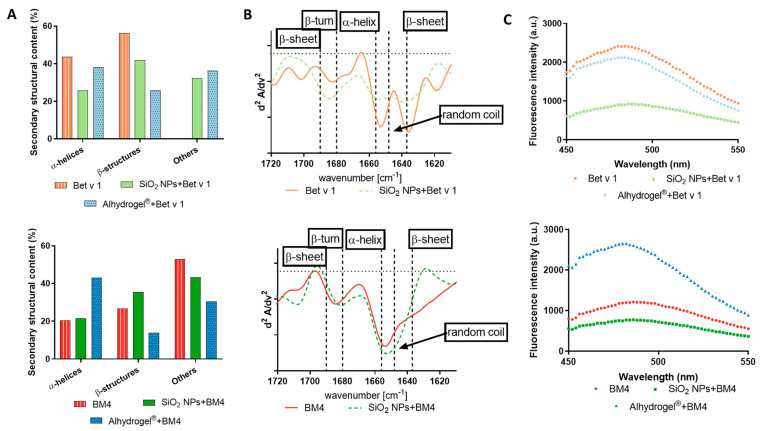
Determination of the fold stability of allergens conjugated to SiO_2_ NPs and Alhydrogel^®^. (**A**) Comparison of the secondary structural contents of the allergens conjugated to particulate systems and unconjugated allergens by CD spectroscopy and (**B**) 2nd derivatives of amide I by Bio-ATR-FTIR spectroscopy, and (**C**) determination of the accessibility of the hydrophobic cavity by ANS spectroscopy. The upper panel indicates samples with Bet v 1 and the lower panel those with BM4.

The proteolytic resistance of allergens depends on their structural integrity; thus, a partial or complete unfolding of the allergen can make it more susceptible to proteolytic enzymes. The proteolytic cleavage sites for Bet v 1 are mostly located within its secondary structures, thus making Bet v 1 relatively proteolytically resistant [40,41]. Therefore, we investigated if the conjugation of the allergens to SiO_2_ NPs or Alhydrogel^®^ changed the conformational stability of both model allergens. From the circular dichroism (CD) spectroscopy data, it was evident that conjugation to SiO_2_ NPs induced conformational changes in the allergens (Figure S5). The deconvoluted CD spectra indicated that upon conjugation to SiO_2_ NPs, the allergen significantly lost its alpha-helical structures, as seen in the case of Bet v 1, whereas in the case of BM4 (partially unfolded allergen), it induced a partial stabilizing effect in the alpha-helical content (Figure 6A). This may be due to the interaction of the positively charged amino acid residues in both the allergens with the negatively charged surface of SiO_2_ NPs. In the case of Alhydrogel^®^, we observed that the alpha-helical structures were relatively stable, whereas there was notable depletion in the beta structures in both model allergens. However, in the case of BM4, an apparent re-folding effect was observed, as indicated by an increase in the alpha-helical content (Figure 6A). Thus, we hypothesize that in a well-folded allergen (Bet v 1), the interaction disrupts the fold stability, while in a molten globule-like allergen (BM4), the interaction with particles partially induces fold stabilization, as indicated by the increase in beta structures in BM4 conjugated to SiO_2_ NPs and alpha structures in Alhydrogel^®^. The distinct stabilizing effects on BM4 by the two types of particles are intriguing and may occur due to the differently charged surfaces of SiO_2_ NPs and Alhydrogel^®^. The observed beta-stabilizing effect in BM4 by SiO_2_ NPs may result from their high curvature and their potential for electrostatic interactions, whereas in Alhydrogel^®^, the interaction stabilizes the alpha-helical content of BM4 with a concomitant decrease in the beta structure content. We observed that the negatively charged SiO_2_ NPs induced a relative destabilization of the alpha-helical structures in both Bet v 1 and BM4, suggesting that the interaction tends to distort the alpha helices in the protein. Conversely, the positively charged Alhydrogel^®^ repels the alpha helices and attracts the beta structures, thereby destabilizing the beta structures and stabilizing the alpha helices. To further confirm this observation, we carried out infrared spectroscopy, which, in general, is more sensitive to the beta structures of proteins. The results additionally verify that the conjugation of allergens to SiO_2_ NPs did not alter the beta structures (Figure 6B). However, in the case of allergens conjugated to Alhydrogel^®^, interpretation of the data was not possible as Alhydrogel^®^ interfered with the results by exhibiting a strong peak similar to the allergen in the amide I region (Figure S6). The anilino-napthalene sulfonic acid (ANS) fluorescence spectroscopy data reveal that conjugation to SiO_2_ NPs decreased the fluorescence intensity at least two-fold, which is an indication that the interaction of the allergens with the SiO_2_ NPs further prevents the accessibility of hydrophobic regions of Bet v 1. Bet v 1 in its native state exhibits a solvent-exposed hydrophobic cavity favoring the binding of ANS [42]. In the case of the molten globule-like allergen (BM4), conjugation to SiO_2_ NPs led to a slight decrease in the fluorescence intensity, whereas in Alhydrogel^®^, the fluorescence intensity increased to a level seen in native Bet v 1, which suggests that conjugation induces a stabilization of the correctly folded allergen (Figure 6C). This may be an indication that SiO_2_ NPs interact with positive charges in the C-terminal alpha helix of Bet v 1 in close proximity to the solvent-accessible hydrophobic cavity.

The conjugation of the well-folded allergen Bet v 1 to SiO_2_ NPs or Alhydrogel^®^ resulted in fold destabilization (at pH 7.4). During the process of allergen processing, these candidates are subjected to further chemical or physical stress. This includes a change in pH (acidic environment in the endolysosomes). It is obvious that the change to a more acidic environment can further pose chemical stress to the allergens and thereby induce further destabilization or unfolding of the allergen, making it more accessible for proteases [43].

### 2.5. Structural Integrity of T Cell and IgE Epitopes

**Figure 7:**
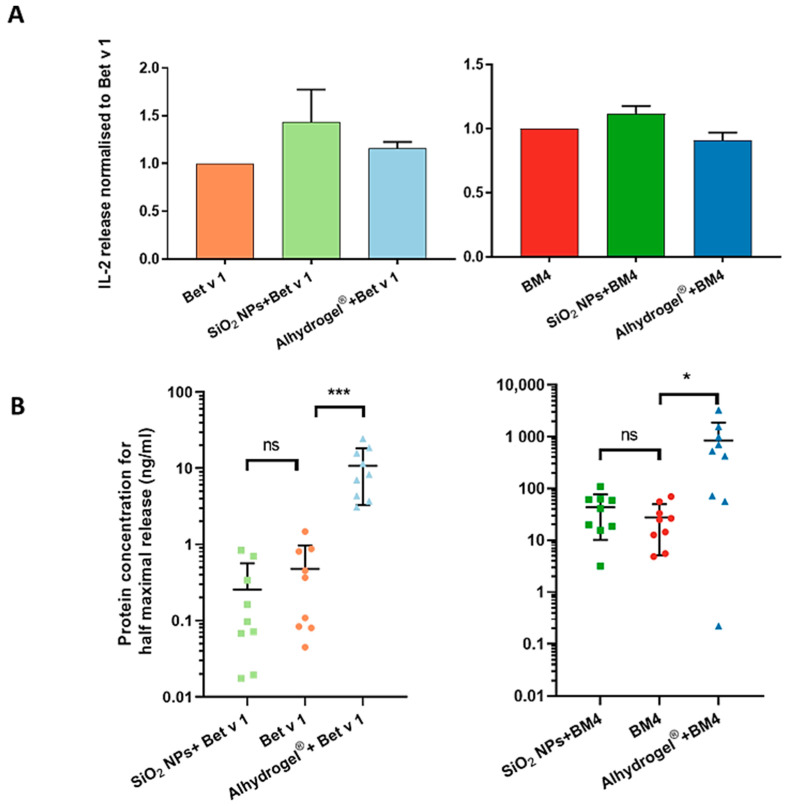
Immunologic properties of conjugated allergens. (**A**) The integrity of the dominant T cell epitope was determined as the concentration of IL-2 (indicator of T cell activation) released from T cell epitope-recognizing T cell hybridoma (panel on the left shows particulate systems with Bet v 1 and that on the right those with BM4). (**B**) The integrity of IgE epitopes was determined by mediator release assay using a humanized rat basophil leukemia cell line (panel on the left shows particulate systems with Bet v 1 and that on right those with BM4). Values are expressed as the protein concentration required to attain half-maximal release compared to the maximum release attained with 10% Triton X-100. Stars indicate the significance of difference in the protein concentration for half-maximal release: *** *p* < 0.0001, * *p* < 0.01.

The conformational stability of allergens has been reported to alter their allergenicity and immunogenicity [35]. We, therefore, sought to investigate if the changes in the fold stability of the allergens upon conjugation resulted in any differences in the immunological properties of the allergens. We determined T cell activation using a dominant T cell epitope recognizing T cell hybridoma by measuring the concentration of secreted interleukin (IL)-2, a cytokine indicative of T cell proliferation, but we could not detect any significant differences between the conjugated and the unconjugated allergens. However, we observed a tendency for an increase in the IL-2 concentration when conjugated to SiO_2_ NPs. This increase most likely resulted from the activation of T cells by SiO_2_ NPs alone (Figure S7). This observation also corresponds to the appearance of more peptides in the immunodominant T cell epitope cluster (Figure 4 and Figure 5). Even though the conjugation affected the 3D fold of the allergens, it did not drastically affect the integrity of T cell epitopes. This confirms the functional integrity of T cell epitopes in both conjugated forms (Figure 7A). Decreased fold stability of an allergen can also lead to the loss of conformational epitopes. This property has been applied for hypoallergenic variants, where the T cell epitope is still intact and functional, whereas conformational B cell epitopes recognized by IgE are reduced [44,45]. We investigated the integrity of IgE epitopes by a mediator release assay using a humanized rat basophil leukemia cell line assay. As shown in Figure 7B, conjugation of both allergens to SiO_2_ NPs did not change their capacity to crosslink cell-bound IgE. However, allergens conjugated to Alhydrogel^®^ significantly reduced their IgE crosslinking capacity compared to the unconjugated allergens. Thus, we could confirm that the surface structure of the SiO_2_ NP-conjugated allergens resembled the native Bet v 1, whereas in Alhydrogel^®^, it was significantly altered or blocked by Alhydrogel^®^ binding. The IgE epitope binding in Bet v 1 has been established to be strongly conformation-dependent [46]. There are also reports on amino acids that may be involved in the recognition of IgE antibodies. This includes amino acids E42, N43, I44, E45, G46, N47, G48, G49, P50, G51 and T52, and R70, D72, H76, I86 and K97 [47,48]. These amino acids are predominantly located in the beta structures of Bet v 1. In the case of SiO_2_ NPs, the beta structures are stabilized, whereas in Alhydrogel^®^, they are distorted and electrostatically attracted, i.e., blocked. It is likely that this distortion, and potential blockage, of beta structures is the reason for the decreased IgE crosslinking.

## 3. Materials and Methods

### 3.1. Patients and Sera

Sera from birch pollen-allergic patients were collected for the mediator release assay and were selected (*n* = 10) based on their allergen-specific IgE reactivity. The procedure was approved by the local ethics committees of the Medical University and General Hospital of Vienna (No. EK1263/2014) and Salzburg (No. 415-E/1398/4-2011).

### 3.2. Synthesis and Physicochemical Characterization of SiO_2_ NPs

SiO_2_ NPs were synthesized by the microemulsion method using tetraethylorthosilicate (TEOS) (Sigma, Darmstadt, Germany) as the precursor, as previously described [49]. A detailed description of the synthesis can be found in the Supplementary Materials File. Alhydrogel^®^ was purchased from Brenntag, Germany. The size distribution, polydispersity index and zeta potential of particulate systems were determined using dynamic light scattering (Malvern Zetasizer Nano ZS, Malvern instruments Ltd., Malvern, UK) and nanoparticle tracking analysis (NanoSight LM10, Malvern instruments Ltd., Malvern, UK). The morphology of the particulate systems was observed by a transmission electron microscope (EM 910, Zeiss, Oberkochen, Germany; JEM F-200, JEOL, Freising, Germany). The average primary particle size of the SiO_2_ NPs was calculated from the TEM images by utilizing 30 particles.

### 3.3. Determination of the Efficiency of Conjugation

Two recombinant pollen allergens, Bet v 1.0101 and BM4 (hypoallergenic variant of Bet v 1), were chosen as the model allergens for this study. Both allergens were produced and characterized in our laboratory according to previously published protocols [24,50]. NPs and Alhydrogel^®^ (2 mg/mL) were incubated with 160 µg/mL of allergens in an isotonic environment by maintaining a pH of 7.4 and a temperature of 4 °C for 17 h on a rotator. After incubation, the samples were centrifuged at 18,000× *g* for 1 h at 4 °C, and the supernatant and pellet were separated. The obtained pellet was then washed with endotoxin-free water to remove the unbound proteins. The protein content of the pellet was determined by SDS-PAGE by successfully separating the allergen bound to the NPs using a reducing buffer, whereas the supernatant was analyzed by colorimetric protein assays, which included Bradford and BCA assays (Sigma-Aldrich, St. Louis, MI, USA). The supernatant was also quantified using SDS-PAGE, and the amount of adsorbed allergen was back-calculated from the value obtained. The identity of the allergens was confirmed using standard molecular weight markers, while the quantity of adsorbed allergens was determined by comparing the intensity of the bands of the samples with the standard allergen concentration after Coomassie brilliant blue R250 staining (Bio-Rad, Irvine, CA, USA). The absolute quantity of adsorbed allergens was calculated using the quantity tool function of Image lab 6.01 software. The efficiency of conjugation was calculated by determining the percentage of allergen bound to the NPs. Furthermore, allergen corona formation was qualitatively confirmed by DLS.

### 3.4. Simulation of In Vitro Endolysosomal Degradation Using Microsomes

The endolysosomal degradation assay was performed with SiO_2_ NPs and Alhydrogel^®^ conjugated to both model allergens and compared to unconjugated allergens, as previously described [37,51]. The samples containing 5 µg equivalent allergen were incubated with 7.5 µg of microsomal extract of JAWS II (American Type Culture Collection, Manassas, VA, USA) in 0.1 M citrate buffer pH 4.8 and 2 mM Dithiothreitol (DTT) for 0, 0.5, 1, 3, 6, 12, 24 and 48 h at 37 °C. At the end of incubation, the digestion was halted by incubation of samples at 95 °C for 5 min. The intact protein after different time points was analyzed by SDS-PAGE and quantitatively determined using Image lab 6.01 software. The proteolytic stability of conjugated allergens was compared with the unconjugated allergens. The quantitative amount of allergen at the zero time point was considered as 100%. The abundance of generated peptides obtained after 1 h of digestion was further analyzed by mass spectrometry (MS) using a Q-Exactive Orbitrap Mass Spectrometer (Thermo Fisher Scientific, Waltham, MA, USA), nanoelectrospray ionization and nano-HPLC (Dionex Ultimate 3000, Thermo Fisher Scientific) [35]. The MS data were qualitatively analyzed using MS Tools software [52]. Furthermore, the generated MS data were quantitatively analyzed by calculating the relative abundance of the generated peptides at 1 h of degradation. This was achieved by clustering the obtained peptide sequences into 8 groups according to their amino acid sequence. For Bet v 1, the clusters are cluster 1: GVFNYETETTSVIPAARLFKAFILD, cluster 2: FKAFILDGDNLFPKVAPQA2, cluster 3: ILDGDNLFPKVAPQAISSVENIEGNGGPGTIKKISFPEGFPFK, cluster 4: YVKDRVDEVDHTNFK, cluster 5: YNYSVIEGGPIGDTLEKISN, cluster 6: GDTLEKISNEIKIVATPDGGSILKISN, cluster 7: KAEQVKASKEMGETL and cluster 8: GETLLRAVESYLLAHSDAYN. Similarly, for BM4, the clusters include cluster 1: GVFNYETETTSVIPAARLFKAFILD, cluster 2: FKAFILDGDNLFPKVAPQA2, cluster 3: ILDGDNLFPKVAPQAISSVENIEGNGGPGTIKKISFPEGFPFK, cluster 4: YVKDRVDEVDHTNFK, cluster 5: YNYSVIEGGPIGDTLEKISN, cluster 6: GDTLEKISNEIKIVATPSGSTIKSISN, cluster 7: KAEQVKASKEMGETL and cluster 8: GETLLRAVESYLLAHSDAYN. The peak areas of each cluster were calculated by summing up the sequences appearing under each cluster, and this was expressed in percentage by counting the total sum of the peptide sequences. 

### 3.5. Evaluation of the Changes in the Protein Structure

The changes in the conformation of the proteins with allergen–(nano)particle conjugation were determined by techniques such as ANS spectroscopy, circular dichroism spectroscopy and Fourier transform infrared spectroscopy (FTIR). The changes in the accessibility of hydrophobic regions of allergens when bound to particles were monitored by using the fluorescent probe ANS. The samples (SiO_2_ NPs and Alhydrogel^®^ conjugated with both model allergens) and controls (unconjugated allergens) at a concentration of 19 µM (90 µL) were incubated with 1 mM of ANS (10 µL). The fluorescence intensity was measured after 30 min using an Infinite M200 Pro plate reader (Tecan, Grödig, Austria). The excitation wavelength was set at 370 nm, and an emission scan was performed from 450 to 550 nm. For CD spectroscopy, samples (Alhydrogel^®^ and SiO_2_ NPs with BM4 and Bet v 1 and unconjugated allergens) were diluted to a concentration of 0.12 mg/mL using 10 mM sodium phosphate buffer, pH 7.4, and the ellipticity was measured with a JASCO J-815 spectropolarimeter fitted with a PTC-423S Peltier-type single position cell holder (Jasco, Tokyo, Japan) over the wavelength range of 190 to 260 nm. The CD spectra obtained were deconvoluted using Bestsel software [53]. The changes in protein structure were confirmed using FTIR where the spectra in the range of the amide I and amide II peaks (1500–1700 cm^−1^) were recorded at a constant temperature (25 °C) using a Bio-ATR II transmission cell adapted to a Tensor II FTIR system (Bruker Optics, Bremen, Germany). Protein concentrations of 1.0 mg/mL were used for the measurement. The analysis of the software was accomplished by using OPUS spectroscopy software 6.0 (Bruker Optics). The second derivative of the amide I vibration was calculated after vector normalization (25 smoothing points) using the Savitzky–Golay algorithm [46].

### 3.6. Determination of the Structural and Functional Integrity of T Cell Epitope by T Cell Activation Assay

To determine the structural and functional integrity of the T cell epitopes of the conjugated allergens to SiO_2_ NPs or Alhydrogel^®^, a T cell activation assay was performed. SiO_2_ NPs and Alhydrogel^®^ bound to both model allergens along with unconjugated allergens, as controls, were incubated with murine bone marrow-derived dendritic cells (BMDCs) from BALB/c mice at a concentration of 10 µg/mL or 5 µg/mL for a defined period of 16 h. After incubation, BMDCs were washed and cocultured with CD4+ T cell hybridoma cells specific for the immunodominant epitope of both proteins (amino acids 142-153) in a ratio of 1:10 for 16 h. The supernatants were harvested, and the concentration of IL-2 release was measured by ELISA (ELISA MAX^TM^ standard set mouse IL-2, Biolegend, CA, San Diego, USA). To avoid the variability within repeated experiments, the concentration of IL-2 release was normalized to unconjugated Bet v 1 and BM4.

### 3.7. Determination of the Structural and Functional Integrity of IgE Epitopes by Mediator Release Assay

To determine the structural and functional integrity of the IgE epitopes of the allergens when conjugated to SiO_2_ NPs or Alhydrogel^®^, a mediator release assay was performed [54]. Human high-affinity IgE receptor (FcεRI)-transfected rat basophilic leukemia cells (RBL-2H3) were sensitized with the sera (containing allergen-specific IgE antibodies) of birch pollen-allergic patients overnight at 37 °C, 5% CO_2_. Sera from birch pollen-allergic patients were selected (*n* = 10) based on their allergen-specific IgE reactivity. All of the patients’ sera were preincubated with AG-8 cells (ATCC, Germany) in order to deactivate the complement system. After incubation, cells were washed with Tyrode’s buffer (9.5 g/L Tyrode’s salts (Sigma), 1 g/L sodium bicarbonate and 0.1% (*w*/*v*) BSA) and incubated with either SiO_2_ NP- or Alhydrogel^®^-bound model allergens as well as the unconjugated allergen controls for 1 h at 37 °C, 5% CO_2_. The concentrations of allergens used for the assay ranged from 10,000 to 0.0001 ng/mL (8 serial dilutions). After the incubation time, the supernatants were collected, and the fluorogenic substrate 4-methyl umbelliferyl-*N*-acetyl-β-glucosaminide (Sigma-Aldrich, St. Louis, MO, USA) was added. The release of β-hexosaminidase into the supernatant cleaves the fluorogenic substrate, leading to fluorescence. The reaction was stopped after 1 h using 0.2 M glycine buffer pH 10.7. The fluorescence intensity was measured at an excitation wavelength of 360 nm and emission at 440 nm, and the percentage of release was calculated by comparing it with the maximum release attained with 10% Triton X 100 (Sigma-Aldrich). The viability of the cells was confirmed with an MTT assay. The results were statistically evaluated by ANOVA followed by a Bonferroni post hoc test. A p value greater than 0.05 was considered as not statistically significant.

## 4. Conclusions

In this study, we utilized an in vitro test strategy that is suitable to analyze antigens at the material interface during the early stage of product development. The strategy comprises biophysical methods and functional assays to understand the structural integrity of proteins, using allergens as models here, at the particle surface. Our findings indicate the dominant role of electrostatic interactions in modulating protein–particle interaction, and we were able to prove that both particulate systems induced structural alterations in the model allergens. These changes significantly altered the immunological response, as confirmed by the biophysical and functional assays. This is a strong indication that the structural stability of antigens should be thoroughly investigated in the initial stages of nanovaccine development. In our study, when we compared the safety of the two particulate systems, the results revealed Alhydrogel^®^ was still a better candidate for allergen-specific immunotherapy, as it exhibited a decreased IgE crosslinking potential, which is an indication of lower adverse effects. Furthermore, it showed better conjugation efficiency putatively due to its nanofibrillar, highly agglomerated and polydisperse state. For SiO_2_ NPs, IgE crosslinking was similar to the unconjugated allergen, thereby indicating a risk of adverse effects during allergen-specific immunotherapy. However, fine-tuning of these NPs through surface functionalization could potentially attenuate this fold destabilization effect, thereby maintaining structural integrity. This could be a desirable property for other types of nanovaccine development. Thus, our early decision toolbox can readily be employed to compare an array of different NP and/or antigen candidates.

In summary, we herein described an early-stage in vitro test strategy applied for nanomaterial candidates for use as adjuvants and/or carriers of allergens, but which can also be applied for other diverse antigens in nanovaccine development, allowing fine-tuning of the nanovaccine properties in order to achieve a desired immune response. This approach, integrated early into the development process, can contribute to the establishment of safe and efficacious nanovaccines in a cost-effective and time-efficient manner and, thus, aid safe-by-design approaches in nanovaccine innovation.

## Figures and Tables

**Table 1:** Physicochemical characterization of the synthesized SiO_2_ NPs and Alhydrogel^®^ by measurement of hydrodynamic size, polydispersity index and zeta potential by DLS (NP concentration of 0.1 mg/mL) and NTA (NP concentration of 0.02 mg/mL).

Sample	Technique	Number Mean Diameter (nm)	PDI	Size (Z Average) (nm)	Zeta Potential (mV)
SiO_2_ NPs	DLS	100.3 ± 3.4	0.025	120.2 ± 1.2	−38.9 ± 2.8
SiO_2_ NPs	NTA	102.4 ± 39.3	-	-	-
Alhydrogel^®^	DLS	585.9 ± 174.2	0.345	1082.0 ± 63.4	+18.0 ± 1.5

**Table 2:** Zeta potential of the particles and particle–allergen conjugates determined by DLS.

Sample	Zeta Potential (mV)
SiO_2_ NPs	−38.9 ± 2.8
SiO_2_ NPs + Bet v 1	−25.7 ± 5.8
SiO_2_ NPs + BM4	−19.2 ± 5.5
Alhydrogel^®^	+18.0 ± 1.5
Alhydrogel^®^ + Bet v 1	+31.2 ± 1.2
Alhydrogel^®^ + BM4	+32.3 ± 1.4

## Data Availability

All data generated or analyzed during this study are included in this manuscript and its supplementary information files.

## Supplementary Materials

The following are available online at https://www.mdpi.com/article/10.3390/ijms221910895/s1.
