# Supplementary material for: Structural Alterations of Antigens at the Material Interface: An Early Decision Toolbox Facilitating Safe-by-Design Nanovaccine Development
Source: Int J Mol Sci. 2021 Oct 8;22(19):10895. doi: 10.3390/ijms221910895 (PMC8509464; doi:10.3390/ijms221910895)
Supplement: Supplementary file 1 [file ijms-22-10895-s001.zip › ijms-1373527-supplementary.pdf]

Supplementary materials for

Structural Alterations of Antigens at the Material Interface: An Early Decision Toolbox Facilitating Safe-by-Design Nanovaccine Development

Litty Johnson, Lorenz Aglas, Wai Tuck Soh, Mark Geppert, Sabine Hofer†, Norbert Hofstätter, Peter Briza, Fatima Ferreira, Richard Weiss, Hans Brandstetter, Albert Duschl, and Martin Himly*

Department of Biosciences, University of Salzburg, Salzburg, Austria

Methods

1. Synthesis of SiO_2 nanoparticles

100 nm silica nanoparticles (NPs) were synthesized in our laboratory using a water-in-oil (W/O) reverse microemulsion method, based on the principles described by Malik *et al.* [1] At room temperature, 15 mL cyclohexane and 3.6 mL n-hexanol were mixed and stirred vigorously (500 rpm) followed by the addition of 3.54 mL Triton X-100. Afterwards, 800 µL dH_2O was added dropwise and the mixture was stirred for 60 min. Then 670 µL TEOS was added dropwise, followed by stirring for 30 min. Subsequently, 240 µL ammonium hydroxide (75% wt H_2O, 25% wt NH_3) was added by gently stirring, continued for 10 min. Soft-stirring (150 rpm) was continued with an interval of 15 min for 3 h. Further 447 µL TEOS was added dropwise with vigorous stirring for 30 min, followed by gently stirring for another 2.5 h. Succeeding this 447 µL TEOS was added again dropwise and stirred vigorously for 30 min. Soft-stirring (150 rpm) was continued with 15/15 min on-off intervals for 16 h. Stirring was again activated, 30 mL acetone

was added and the reaction mix was sonicated for 10 min in a water bath sonicator (Elma, Singen, Germany). The mixture was transferred into two 50 mL Greiner tubes and centrifuged at 3260 *g* for 4 h. Supernatant was discarded and 10 mL acetone were added in each tube. Water bath sonication was applied for 30 min. Both samples were pooled. Centrifugation at 3260 *g* for 1 h was applied, supernatant was discarded, and 15 mL acetone was added, followed by sonication. These steps were repeated three times, with acetone replaced by 10 mL ethanol in the final step. Further storage was done at 4°C.

2. Characterization of SiO_2 NPs and Alhydrogel®

Nanoparticle Tracking Analysis (NTA)

Initially the hydrodynamic particle size of SiO_2 NPs was investigated by NTA. The stock of particles was diluted to obtain a concentration of 0.02 mg/ml in water and injected into the sample insertion cell of NanoSight LM10 (Malvern Instruments Ltd, Malvern, UK). The measurement was performed by recording five videos of each 30 s duration. On average 73.2+/-3.9 particles per frame were measured with a camera level of 11 and by maintaining temperature of 25ºC. Independent measurements were performed with three individual samples.

Dynamic Light Scattering (DLS)

The particle size, polydispersity index and zeta potential of SiO_2 NPs and Alhydrogel® was measured using DLS (Malvern Zetasizer Nano ZS, Malvern instruments Ltd, Malvern, UK). The stock of particles was diluted to obtain a concentration of 0.1 mg/ml in water and the measurements were carried out in a DTS1070 capillary cell (Malvern instruments, Malvern, UK).

Transmission Electron Microscopy (TEM)

For the sample preparation for transmission electron microscopy 2 µL of a diluted aqueous particle dispersion (0.01 mg/mL for SiO_2 NPs; 0.005 mg/ mL for Alhydrogel®)

was applied on a Formvar coated-copper grid (SiO_2 NPs), lacey carbon-coated Cu grid (Alhydrogel®) and air-dried. The samples were then analyzed using the EM 910 (Zeiss, Oberkochen, Germany (SiO_2 NPs)) operated at 120 kV and JEM F-200 (JEOL, Freising, Germany (Alhydrogel®)) operated at 200 kV.

3. Stability of allergen particle conjugation

The stability of allergen-particle conjugation was determined by the estimation of the amount of allergen bound to the NPs in the degradation medium at different times of incubation. SiO_2 NPs and Alhydrogel® conjugated with both the model allergens were incubated in the degradation medium (0.1 M sodium acetate pH 5, 0.1 M sodium chloride, 5 mM ethylenediaminetetraacetic acid (EDTA) and 2 mM dithiothreitol (DTT) for 0, 0.5, 1, 3, 6, 12, 24 and 48 h at 37°C. At different time points, the pellet and supernatant were separated and the protein content in the pellet was determined by SDS-PAGE upon applying a previously published extraction protocol. [2] The percentage of protein still bound to the SiO_2 NPs and Alhydrogel® after different incubation times was calculated to determine the stability of allergen binding.

4. Enzyme activity assay

Enzymatic activity assays were performed to determine a potential inhibition of rCathepsin S activity by SiO_2 NPs or Alhydrogel®. To determine this, 5 µg allergen bound to NPs and equivalent amounts of NPs without allergen were incubated with 50 µM Z-VVR-AMC (fluorogenic substrate) in the assay buffer (0.1 M sodium acetate pH 5, 0.1 M sodium chloride, 5 mM EDTA and 2 mM DTT) with 10 nM concentration of rCathepsin S to a total volume of 50 µl in a 96 well half area black flat bottom plate (Corning Inc., Corning, USA) at 37°C. The fluorescence intensity was measured using an Infinite M200 pro plate reader (Tecan, Groedig, Austria) at the excitation and

emission wavelengths of 380 and 460 nm, respectively, for a duration of 15 min with a time interval of 30 sec.

5. Simulation of *in vitro* endolysosomal degradation using recombinant human cathepsin S

The expression and purification of recombinant human cathepsin S (rCathepsin S) was performed according to previously reported protocols.[3] The samples (SiO_2 NPs and Alhydrogel® conjugated with both the model allergens) containing 20 µM equivalent allergen were incubated in 0.1 M sodium acetate pH 5, 0.1 M sodium chloride, 5 mM EDTA and 2 mM DTT with 1 µM concentration of rCathepsin S for 0, 0.5, 1, 3, 6, 12, 24 and 48 h at 37°C. At the end of each incubation, the enzymatic degradation was stopped by the addition of 50 µM E64 (cathepsin S inhibitor). The intact protein at different time points was analyzed by SDS-PAGE and quantitatively determined using Image lab 4.01 Software.

Figures

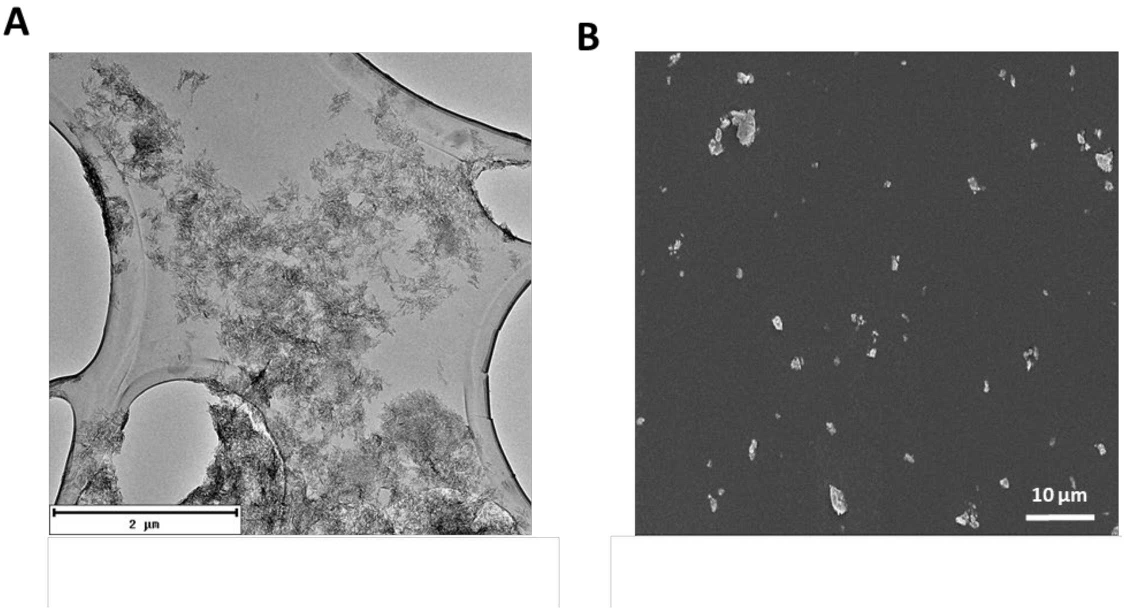

Figure S1. Transmission electron microscopy (TEM) image of synthesized Alhydrogel® **(A)** and scanning electron microscopy (SEM) image of Alhydrogel® **(B)**.

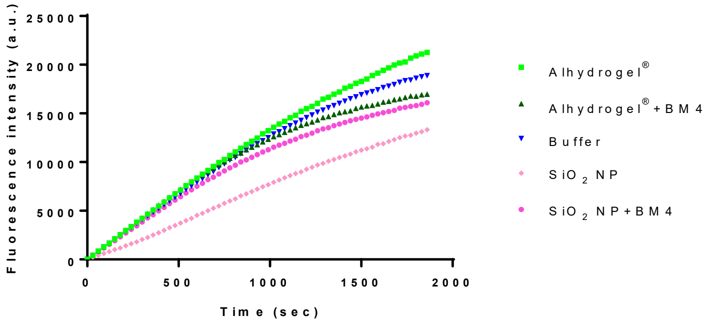

Figure S2. Enzymatic activity of microsomal extracts in the presence of allergen conjugated to particulate systems. A broad cathepsin fluorogenic substrate (VVR-AMC) was used to access the major cysteine cathepsins activities.

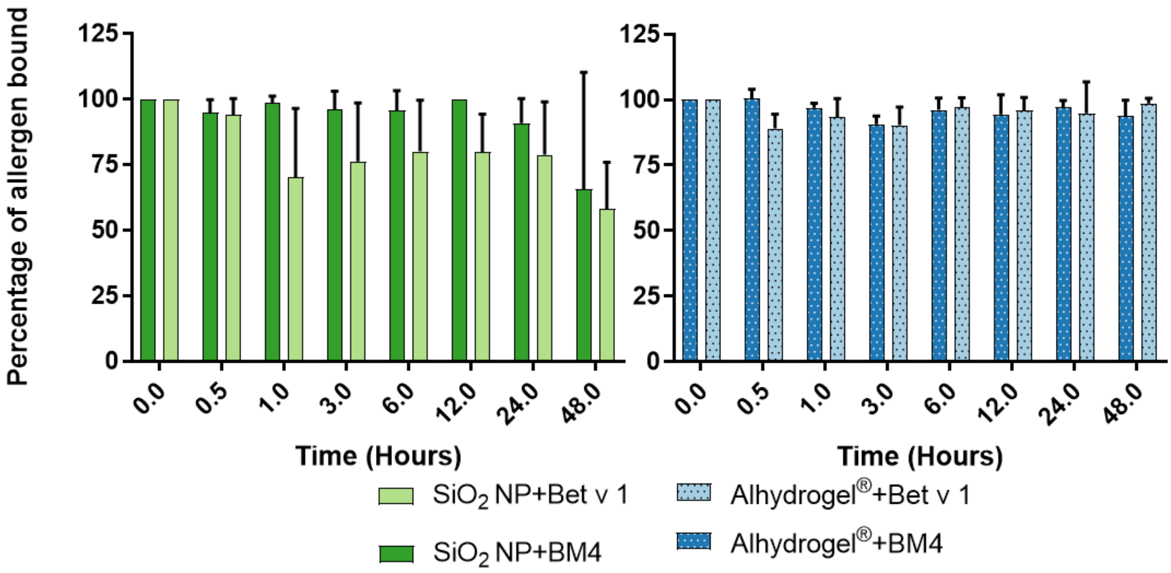

Figure S3. Determination of the stability of allergen conjugations after incubation in the degradation medium at 37˚C for different time points (0, 0.5, 1, 3, 6, 12, 24, 48 h) by SDS-PAGE calculated by the densitometric analysis of the proteins using Image lab 4.01software.

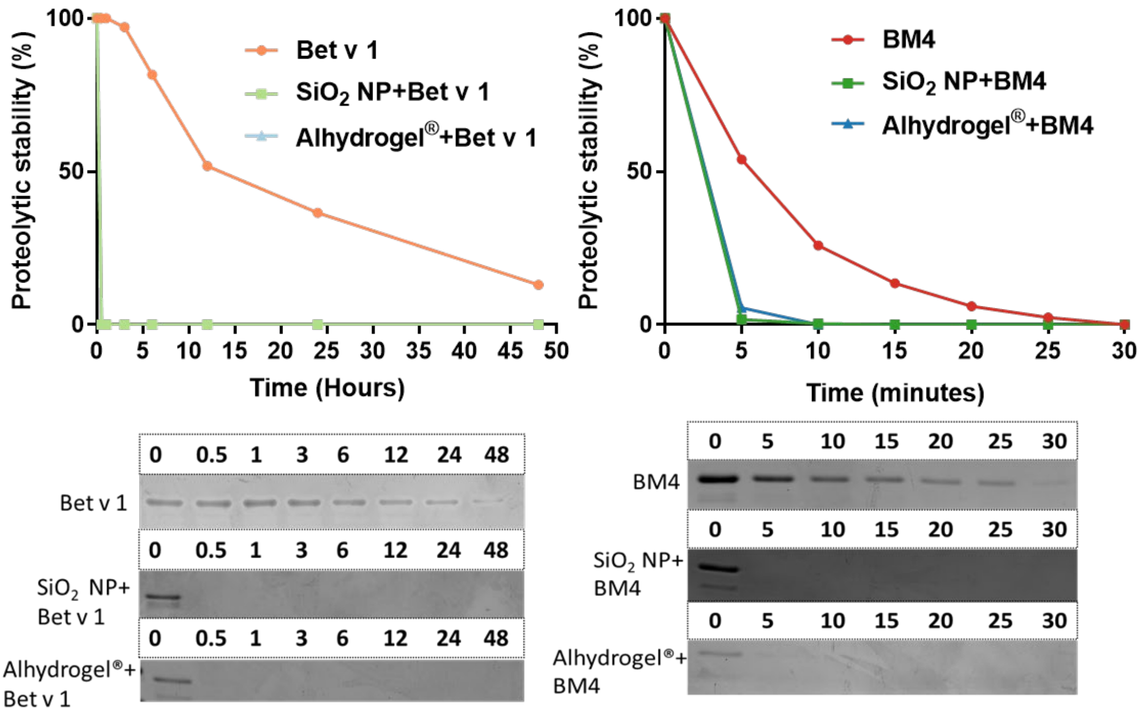

Figure S4. Comparison of the kinetics of proteolytic stability of SiO_2 NPs-, Alhydrogel®-conjugated allergen and unconjugated allergen by using rCathepsin S at 37˚C for different time points (0, 0.5, 1, 3, 6, 12, 24 and 48 h).

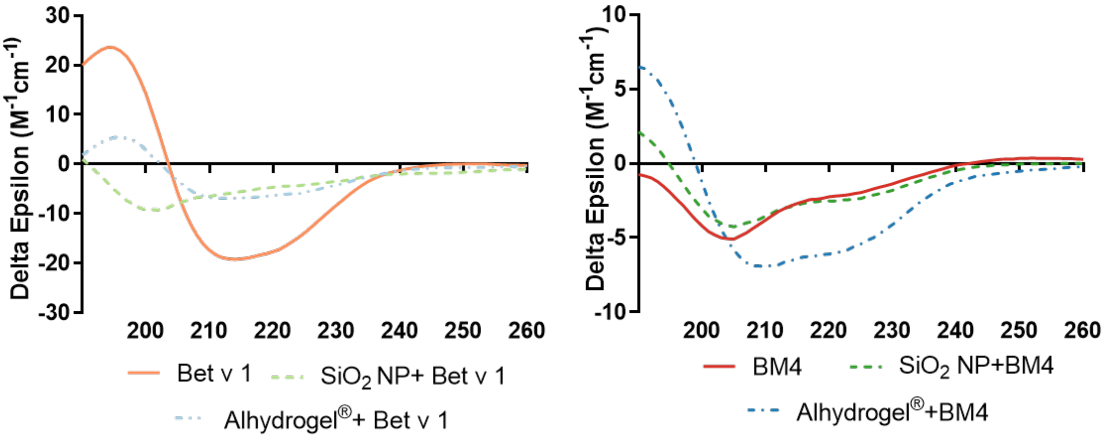

Figure S5. CD spectra of the allergens (Bet v 1 and BM4) conjugated to SiO2 NP and Alhydrogel® and unconjugated allergen at pH 7.4. The spectra have been smoothed using smooth function of GraphPad prism 8.

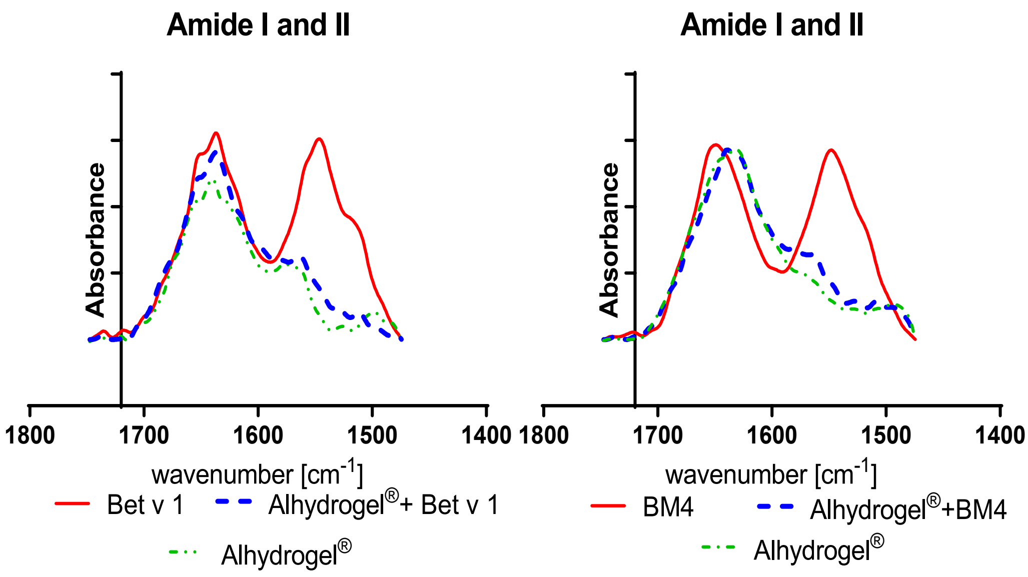

Figure S6. Amide I and II spectra of allergens conjugated to Alhydrogel® obtained from the FTIR spectroscopy.

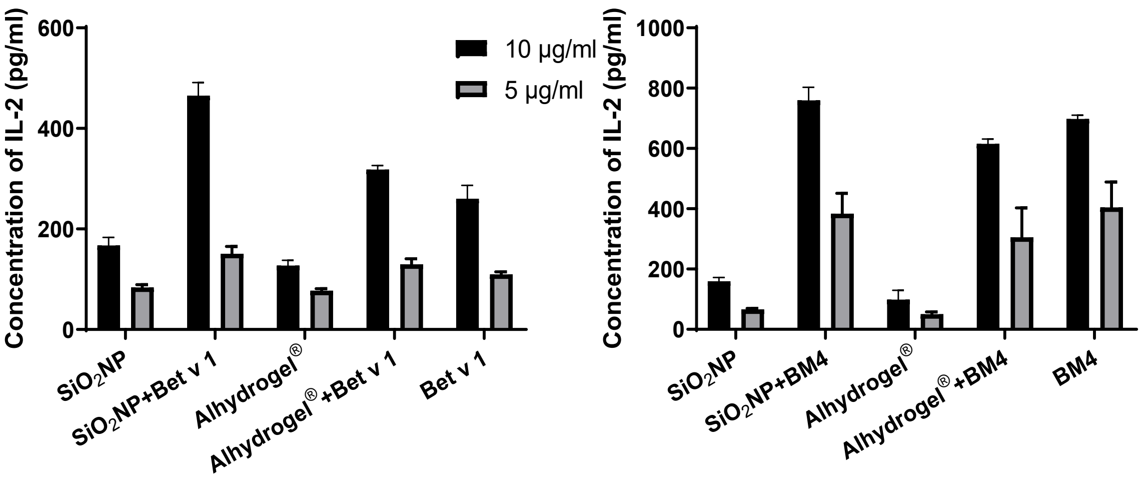

Figure S7. A representative result showing the concentration of IL-2 release when incubated with a concentration of 5 and 10 μg/ml equivalent allergen to determine the functional integrity of the T cell epitope

References

1. Malik, M.A.; Wani, M.Y.; Hashim, M.A. Microemulsion method: A novel route to synthesize organic and inorganic nanomaterials: 1st Nano Update. *Arabian J. Chem.* **2012**, *5*, 397-417.

2. Zhu, D.; Huang, S.; McClellan, H.; Dai, W.; Syed, N.R.; Gebregeorgis, E.; et al. Efficient extraction of vaccines formulated in aluminum hydroxide gel by including surfactants in the extraction buffer. *Vaccine* **2012**, *30*, 189-94.

3. Freier, R.; Dall, E.; Brandstetter, H. Protease recognition sites in Bet v 1a are cryptic, explaining its slow processing relevant to its allergenicity. *Sci. Rep.* **2015**, *5*, 1-9.
